# Allergy testing at OLCHC

**DOI:** 10.1186/2045-7022-5-S3-O19

**Published:** 2015-03-30

**Authors:** Cathryn O'Carroll, John Brady, Joe McNamara, Philip Mayne, Aideen Byrne

**Affiliations:** 1 OLCHC, AMNCH, Dublin, Ireland

## Background

Allergy is a growing problem in the western world. However, education of health care workers in the field of allergy has not kept up with clinical need. There has been much evidence that medical practitioners use diagnostic tests inaccurately, declaring food allergy where there is none. Removal of food from a patient's diet, especially a child's, has significant nutritional implications that can be irreversible and have a lifelong effect. Furthermore the cost of inappropriate tests is escalating. Guidelines have recently been set indicating that allergy focused history must guide all allergy orders: http://www.ifan.ie.

## Aims of this audit

1. To optimise the allergy testing service provided for both clinicians and patients.

2. To examine whether allergy testing was in line with current guidelines.

3. To look for areas where cost efficiency could be improved.

4. To gather local data that can be incorporated into education sessions for clinicians.

## Methodology

All sIgE tests ordered through the laboratory at Our Lady's Children's Hospital, Crumlin, from May 8th to Nov 7th 2013, were made available in the form of an excel spread sheet. The outcomes of 4 tests were evaluated, according to age of patient and department from where order originated.

• House Dust Mite sIgE

• Grass sIgE

• Common food panel

• Fruit sIgE

## Results

1. *HDM sIgE*

• 679 tests were ordered

• 25% of all tests were ordered on those <2 yrs and only 15/153 (10%) were positive.

**Table 1:** 

	Negative	Positive
< 1yr	77 (90%)	8(10%)

1-2yr	76	7

Average age	3.8+/-3.6	6.3 +/-4.2

2. *Grass pollen sIgE*

• 638 tests were ordered

• 73% were negative

Positivity increased with age consistent with all international data.

**Table 2:** 

	Negative	Positive
Average age	3.8 + /-3.8	7.4 +/-4.2

Median age	3yr	7yr

< 1yr	83(100%)	0

1-2yr	83 (98%)	2

**Figure 1:**
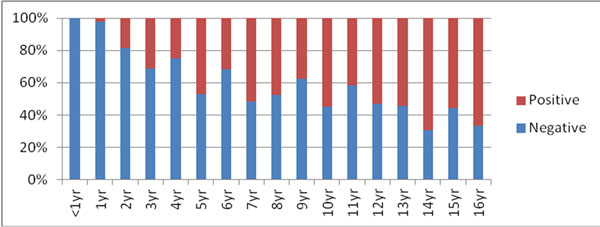


3. *Testing for fruit allergy*

• All of these tests were negative

**Table 3:** 

Food	Total	Positive		Origin
Orange	15	0		80% CPs

Strawberry	11	0		56%CPs

4. *The common food panel:*

• 519 tests were performed

• 59% of these were entirely negative

**Figure 2:**
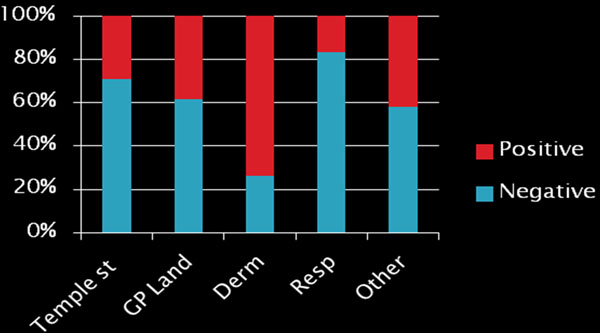


**Table 4:** 

Conclusions	Recommendations	Target date	Responsible person
Clinicians have a poor understanding of how to use sIgE testing	Formal lecture to NCHDs	January 2014 (achieved)	Dr Aideen Byrne

Tests are being run inappropriately with considerable cost implications	Establish hospital protocol for ordering of IgE tests	May 2014	Dr Cathryn O Carroll Dr Aideen Byrne

Laboratory staff are unclear how to judge appropriateness of test order.	Establish clear guidelines for laboratory staff	May 2014	Joe McNamara

Specific IgE testing for aeroallergens being ordered in wrong population	Orders for sIgE to HDM and Grass pollen not to be accepted under 2 years	May 2014	Aideen Byrne Joe McNamara

